# Limited Cross-Protection against Infectious Bronchitis Provided by Recombinant Infectious Bronchitis Viruses Expressing Heterologous Spike Glycoproteins

**DOI:** 10.3390/vaccines8020330

**Published:** 2020-06-22

**Authors:** Sarah Keep, Samantha Sives, Phoebe Stevenson-Leggett, Paul Britton, Lonneke Vervelde, Erica Bickerton

**Affiliations:** 1 The Pirbright Institute, Pirbright, Surrey GU24 0NF, UK; sarah.keep@pirbright.ac.uk (S.K.); phoebe.stevenson-leggett@pirbright.ac.uk (P.S.-L.); paul.britton@pirbright.ac.uk (P.B.); 2 Division of Infection and Immunity, The Roslin Institute and Royal (Dick), School of Veterinary Studies, University of Edinburgh, Easter Bush EH25 9RG, UK; samantha.ellis@roslin.ed.ac.uk (S.S.); lonneke.vervelde@roslin.ed.ac.uk (L.V.)

**Keywords:** infectious bronchitis virus, coronavirus, reverse genetics, recombinant vaccine, spike glycoprotein, heterologous challenge, cross-protection

## Abstract

*Gammacoronavirus* infectious bronchitis virus (IBV) causes an economically important respiratory disease of poultry. Protective immunity is associated with the major structural protein, spike (S) glycoprotein, which induces neutralising antibodies and defines the serotype. Cross-protective immunity between serotypes is limited and can be difficult to predict. In this study, the ability of two recombinant IBV vaccine candidates, BeauR-M41(S) and BeauR-4/91(S), to induce cross-protection against a third serotype, QX, was assessed. Both rIBVs are genetically based on the Beaudette genome with only the S gene derived from either M41 or 4/91, two unrelated serotypes. The use of these rIBVs allowed for the assessment of the potential of M41 and 4/91 S glycoproteins to induce cross-protective immunity against a heterologous QX challenge. The impact of the order of vaccination was also assessed. Homologous primary and secondary vaccination with BeauR-M41(S) or BeauR-4/91(S) resulted in a significant reduction of infectious QX load in the trachea at four days post-challenge, whereas heterologous primary and secondary vaccination with BeauR-M41(S) and BeauR-4/91(S) reduced viral RNA load in the conjunctiva-associated lymphoid tissue (CALT). Both homologous and heterologous vaccination regimes reduced clinical signs and birds recovered more rapidly as compared with an unvaccinated/challenge control group. Despite both rIBV BeauR-M41(S) and BeauR-4/91(S) displaying limited replication in vivo, serum titres in these vaccinated groups were higher as compared with the unvaccinated/challenge control group. This suggests that vaccination with rIBV primed the birds for a boosted humoral response to heterologous QX challenge. Collectively, vaccination with the rIBV elicited limited protection against challenge, with failure to protect against tracheal ciliostasis, clinical manifestations, and viral replication. The use of a less attenuated recombinant vector that replicates throughout the respiratory tract could be required to elicit a stronger and prolonged protective immune response.

## 1. Introduction

Infectious bronchitis virus (IBV) is a *Gammacoronavirus* with a positive sense single-stranded RNA genome, consisting of a large replicase gene encoding 15 non-structural proteins (nsps); the structural genes including spike (S), membrane (M), envelope (E) and nucleocapsid (N); and the accessory genes including 3a, 3b, 4b, 4c, 5a, and 5b [1]. IBV is the aetiological agent of infectious bronchitis (IB), which is a disease of domestic fowl that is of both welfare and economic importance to the global poultry industry. Infected chickens display clinical signs including snicking, rales, watery eyes, nasal discharge, and lethargy; they exhibit reduced weight gain and impaired egg laying in terms of both quantity and quality [2,3]. In addition, infected birds are susceptible to secondary bacterial infections as a result of tracheal ciliostasis and immunosuppression [4,5].

Several serotypes and genotypes of IBV co-circulate in poultry flocks. The viral serotype is determined by the major structural protein, spike (S), a type 1 glycoprotein consisting of two subunits; the globular S1 subunit that mediates receptor-binding and the stalk-like S2 subunit that facilitates virus-to-cell and cell-to-cell fusion [6,7]. The majority of neutralising antibodies are targeted towards the S1 subunit, as this contains the receptor-binding domain [8,9,10]. The S2 subunit is more highly conserved than the S1 and also contains immunogenic regions, playing a role in both the generation of a protective immune response [6,11,12] and determining host tropism [13,14]. Several studies, including those utilising recombinant IBV to express full or partial S glycoproteins, have demonstrated the capability of the S protein to induce a degree of a protective humoral immune response against homologous challenge [11,12,15,16,17,18,19] and heterologous challenge [16,20,21]. 

The real challenge in the control of IBV, however, is that vaccination against one serotype often offers limited cross-protection against another serotype. There is often a poor relationship correlation between the level of protection afforded by a vaccine and the level of homology between S glycoproteins from challenge and vaccine strains [22]. Although the mechanisms behind cross-protection still remain to be elucidated, cross-protection can be evident with unrelated vaccine and challenge strains [16,21,22,23,24,25,26]. The application of commercial vaccines of two serotypes, Ma5 and 4/91, against challenge with a third serotype, QX, indicated induction of protective immune response against QX, as the challenge virus was not re-isolated from the trachea [21]. This induction of cross-protective immunity occurred, despite the fact that there was a low genetic relationship between the hypervariable region of the S1 subunit of Ma5 and QX (77.1%) and of that between 4/91 and QX (81%) [21]. Conversely, studies have demonstrated limited cross-protection between strains with high sequence homology [23,27,28]. Despite 95% sequence homology between the S glycoproteins of two viruses belonging to the Massachusetts serotype, vaccination with IBV Beaudette cannot confer protection against a M41-CK challenge [15]. Collectively, this suggests that a small number of virus neutralising epitopes play a disproportionate role in cross-protection, which can make it difficult to predict effective vaccine strategies [22]. 

Effective vaccination against IBV is proving to be an increasing challenge due to the emergence of novel strains, such as QX, first isolated in China, in 1995 [29], and subsequently spread worldwide [30,31,32]. Whilst some strains of IBV have spread worldwide, others have emerged but remained limited to distinct geographical areas [33,34]. Commercial live IBV vaccines are generated through multiple passages of a pathogenic field isolate through embryonated hen’s eggs with the aim of generating a virus that is attenuated but still immunogenic [35,36]. However, attenuation by this method is unpredictable and results in different patterns of genomic variations [37]. Given the nature of this process, it is challenging to readily respond to emerging strains in a time appropriate manner. The development of reverse genetic systems for IBV [38,39,40] has opened the possibility of using recombinant IBVs (rIBVs) as vaccines that can be rationally designed and readily manufactured, removing the cumbersome trial and error prone method of attenuation by multiple passages through embryonated hens’ eggs [37]. In this study, we investigated the use of two rIBVs, BeauR-M41(S) and BeauR-4/91(S), genetically based on the Beaudette genome with only the S gene derived from a different strain, either M41 or 4/91. Both rIBVs have been shown previously to induce partial protection against homologous and heterologous challenge [15,16], but not against heterologous challenge with the QX strain. Here, birds received a primary vaccination of either BeauR-M41(S) or BeauR-4/91(S), and then received a boost with a secondary vaccination of either BeauR-M41(S) or BeauR-4/91(S). The effects of the order in which both vaccines were given on the level of protection and the humoral responses were also investigated. Therefore, through these rIBVs we were able to assess the level of cross-protection induced by M41 and 4/91 S glycoprotein against a heterologous QX challenge. 

## 2. Materials and Methods 

### 2.1. Ethical Statement

All animal experimental protocols were carried out in strict accordance with the UK Home Office guidelines and under license granted for experiments involving regulated procedures on animals protected under the UK Animals (Scientific Procedures) Act 1986. The experiments were performed in The Pirbright Institute Home Office licensed (X24684464) experimental animal house facilities and were approved by the animal welfare and ethical review committee under the terms of reference HO-ERP-01-1.

### 2.2. Cells and Viruses 

Primary chicken kidney (CK) cells were prepared from kidneys extracted from two- to three-week-old specific-pathogen-free (SPF) Rhode Island Red (RIR) chickens [41]. All cell cultures were maintained at 37 °C and 5% CO_2_. Primary tracheal organ cultures (TOCs) were prepared from 19-day-old RIR SPF embryos [42]. Cultures were maintained at 37 °C, rotating at 7–8 revolutions per hour. Recombinant IBV Beau-R and vaccine viruses, rIBV BeauR-M41(S) and BeauR-4/91(S), have been described previously [15,16,38,43]. Both vaccine viruses contain the signal sequence, the ectodomain and the transmembrane domain from either M41-CK (GenBank accession number X04722) or 4/91(UK) (GenBank accession number JN192154). The cytoplasmic tail had been derived from the Beau-R backbone (GenBank accession number AJ311317) and was maintained to preserve interactions with Beaudette-derived viral proteins. The virulent challenge strain QX L1148 [31] (GenBank accession number KY933090) was a gift from Professor Richard Jones at the University of Liverpool. All viruses were propagated in 10-day-old SPF RIR embryonated hens’ eggs and BeauR-M41(S) was titrated in triplicate in CK cells. As neither IBV QX nor rIBV BeauR-4/91(S) can replicate in cell culture [14,16], they were titrated in TOCs, as previously described [15]. The titres were displayed as the number of plaque-forming units (PFU) per ml or as 50% (median) ciliostatic dose (CD50) per mL.

### 2.3. In Vivo Characterisation 

**Figure 1:**
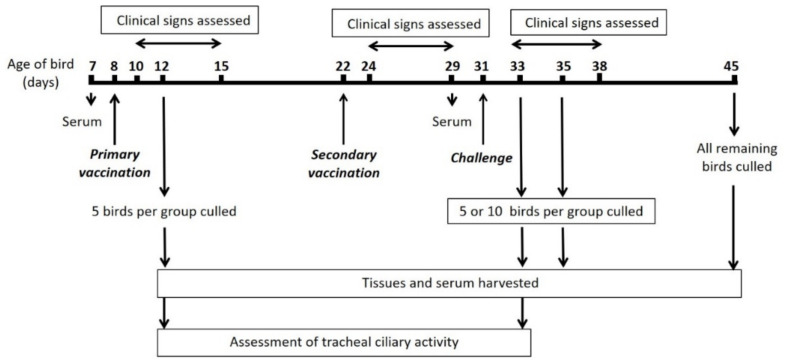
Schematic detailing protocol for the in vivo heterologous vaccine-challenge experiment. Groups of eight-day-old specific-pathogen-free (SPF) Rhode Island Red (RIR) chickens received a primary vaccination of BeauR-M41(S), BeauR-4/91(S), or phosphate buffered saline (PBS). Two weeks (14 days) later, birds received a second vaccination of either BeauR-M41(S), BeauR-4/91(S), or PBS. Nine days post-secondary vaccination (dpsv), birds were challenged with QX or mock challenged with PBS. Clinical signs were assessed for both post-vaccination and post-challenge birds. At defined intervals, randomly chosen birds were culled from each group and a variety of tissues harvested. Serum was collected pre-vaccination, post-vaccination (pre-challenge), and post-challenge. Tracheal ciliary activity was assessed at 4 days post-primary vaccination (dppv) and 4 days post-challenge (dpc). The experiment ended at 14 dpc with all remaining birds culled.

SPF RIR chickens were housed in raised floor pens held within separate positive-pressure HEPA-filtered rooms in groups of 20, 30, or 35 birds. The experimental design is detailed in Figure 1. Chickens were randomly assigned to one of six groups (Table 1), and the sex of the birds was not taken into consideration. Eight-day-old chicks were inoculated (classified as primary vaccination) with 10^4^ PFU of BeauR-M41(S) or the equivalent dose (as determined by a standard curve comparing titre of infectious virus quantified by plaque assay or ciliostatic dose) of 3 log10 CD50 BeauR-4/91(S) in 0.1 ml phosphate buffered saline (PBS) which was split between conjunctival and intranasal routes. Mock vaccinated (control) chickens were inoculated via the same route with 0.1 ml PBS. Fourteen days after the primary vaccination (dppv), chickens received a second vaccination of BeauR-M41(S), BeauR-4/91(S), or PBS which was administered in the same dose and manner as the primary vaccination. Nine days post-secondary vaccination (dpsv), a challenge dose of 3 log10 CD50 QX or 0.1 ml PBS was administered via the same route (Table 1). Birds were monitored for clinical signs including snicking, rales, watery eyes, and nasal discharge from 3–7 days after each inoculation with IBV, as previously described [44]. 

On specific dppv and days post-challenge (dpc), five or 10 randomly selected chickens were culled by cervical dislocation (Table 1). A sample size of five birds was used for the mock control group at all time-points and all vaccinated groups at 4 dppv. This sample size of five birds and 10 tracheal rings per bird was calculated to detect a significant difference in mean ciliary activity of 25% (95% certainty, 80% power, two-sided) between groups post IBV infection [45]. To increase statistical power for the detection of differences in protection levels between vaccinated groups, the sample size was increased to 10 birds post-challenge. A panel of tissues was collected and stored in RNAlater (Ambion, Austin, Texas, United States) or PBS depending on the downstream analysis of viral RNA load and infectious viral load, respectively. Tissues collected included the following: Harderian gland, lower eyelid containing the conjunctiva-associated lymphoid tissue (CALT), and trachea. Blood samples were collected at specific time-points and clarified by low speed centrifugation at 700× *g* for 5 min to collect serum.

### 2.4. Ciliary Activity Assay

Tracheas were removed from five randomly selected chickens per group at 4 dppv and from five or 10 chickens per group at 4 dpc (Table 1). Ciliary activity was assessed as previously described [22,23]. Briefly, 10 one mm rings were cut from each trachea, three from the top, four from the middle, and three from the bottom. Each ring was assessed for ciliary activity and scored using the following system: 0 (no ciliated cells beating, complete ciliostasis), 25, 50, 75, and 100% of cilia beating. The average (mean) of 10 rings was calculated and presented as % ciliary activity. The assessor was blinded to the experimental group. A bird was considered to be protected if 50% or more ciliary activity was retained in nine of 10 tracheal rings; and this was needed in 80% of the group [46]. The remaining trachea was stored in RNA later or in PBS. 

### 2.5. Assessment of Infectious Virus in Tracheal Samples 

Tracheal rings used for the ciliary activity assay at 4 dppv and 4 dpc were homogenised in 500 µL PBS using a Tissuelyser II (Qiagen, Hilden, Germany), producing tissue-derived supernatant that was subsequently clarified by low speed centrifugation. To determine whether infectious virus was present in the trachea at 4 dppv, 10-day-old SPF RIR embryonated hens’ eggs were inoculated with 100 µL of tracheal tissue-derived supernatant. Allantoic fluid was harvested 48 hours post infection (hpi) and assessed for viral presence by RT-PCR using primers specific for the 3ʹ UTR, as previously described [44]. Tissue-derived supernatant from tracheas collected at 4 dpc was titrated in ex vivo TOCS as previously described [15]. The dose required to cause 50% ciliostasis (CD50) was calculated according to the Reed and Muench method [47].

### 2.6. Infectious Bronchitis Virus ELISA 

Serum samples collected pre-challenge, 7 dpsv, and at 4 and 14 dpc were assessed with a commercial IBV antibody test kit (BioChek BV, Reeuwijk, The Netherlands), coated with inactivated whole IBV antigen. The 7 dpsv sera were diluted to 1:80. For determining titre endpoints, the 4 and 14 dpc sera were two-fold serially diluted in the range 1:80–1:2560, prior to incubation. After sample incubation, the remaining steps were followed directly according to the manufacturer’s instructions. The sample/positive (S/P) ratio was determined by the following equation: (Mean sample − Mean Kit Negative)/(Mean Kit positive − Mean Kit Negative). The S/P ratios above 0.2 were considered to be positive for IBV antibodies. Polyclonal chicken serum raised against IBV QX was included on each independent test plate as a reference control (kindly gifted by J.J. de Wit, GD Animal Health).

### 2.7. Detection of Viral RNA

Total RNA was isolated from Harderian gland and CALT using the RNeasy^®^ Mini Kit and DNase-treated following the manufacturer’s instructions (Qiagen). cDNA was synthesised from 1 µg of tRNA using Superscript IV Reverse Transcriptase (Invitrogen by Life Technologies, Carlsbad, California, United States) with a random oligo primer as per the manufacturer’s instructions. For quantification of viral RNA load, qPCR was performed using the Taqman Universal PCR Master Mix (Applied Biosystems, Foster City, California, United States) with primers and probes specific to the 5ʹ UTR region [48]. The RT-qPCR data was analysed in the same manner as previously described [12].

### 2.8. Statistics

Serum antibody levels, ciliary activity, infectious viral load, and viral RNA load were tested for normality. Differences between ciliary activities, and pre-challenge serum titres were evaluated by non-parametric Kruskal–Wallis adjusted post hoc for multiple comparisons, Mann–Whitney, and Dunn’s, respectively. Differences between post-challenge serum titres were assessed by two-way ANOVA, adjusted for post hoc analysis Tukey’s pairwise comparisons. The differences between the viral load RT-qPCR data and infectious viral load titres were statistically evaluated by the parametric one-way ANOVA test adjusted for post hoc analysis Tukey’s pairwise comparison. Differences in viral titres in response to serum neutralisation were evaluated using a two-way ANOVA with a Tukey test for multiple comparisons. Mean and standard error of the mean (SEM) values are displayed.

## 3. Results

### 3.1. Confirmation that the S Gene from a Pathogenic Strain Does Not Confer Virulence to a Non-Pathogenic Strain 

**Figure 2:**
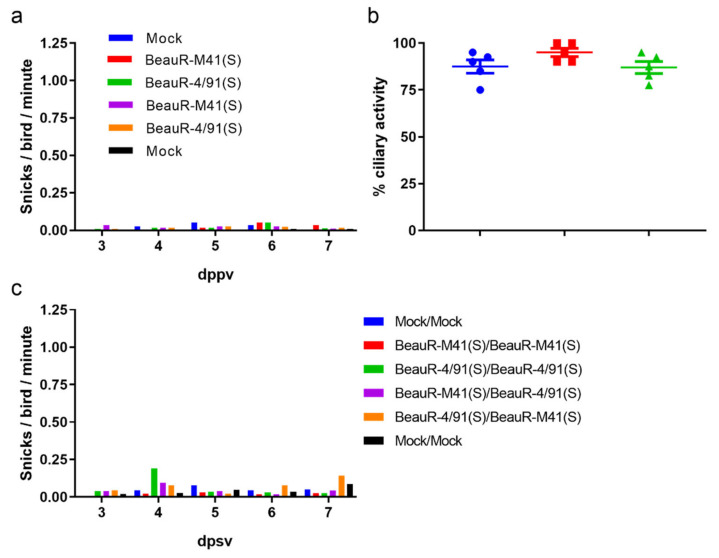
The S gene from a pathogenic strain does not confer virulence to a non-pathogenic strain. SPF birds, at eight days of age, were vaccinated with either BeauR-M41(S), BeauR-4/91(S), or PBS for mock vaccination. (**a**) The number of snicks in each group was assessed from day three to seven days post-primary vaccination (dppv). The numbers of snicks were independently counted over a two-minute period by two or three persons with the average (mean) of these scores presented; (**b**) Trachea was harvested from five randomly sampled birds four dppv. Each trachea was sectioned in 10 × 1 mm rings and the ciliary activity of each ring was assessed by light microscopy and the percentage activity calculated. Plotted points represent individual birds and the mean activity of the 10 rings assessed. Error bars represent standard error of the mean (SEM). Statistical differences were evaluated using a Kruskal–Wallis test followed by a post hoc Mann–Whitney test corrected for multiple comparisons; no differences were identified; (**c**) Fourteen days post-primary vaccination, birds received a secondary vaccination of either BeauR-M41(S), BeauR-4/91(S), or PBS for mock vaccination. The numbers of snicks in each group were independently counted over a two-minute period by two or three persons from day three to seven post-secondary vaccination (dpsv) with the average (mean) of these scores presented. Snicking post-secondary vaccination was comparable between the vaccinated groups and the mock vaccinated groups.

To elucidate whether homologous or heterologous vaccination, with rIBV expressing the S gene from either 4/91 or M41-CK, could cause any clinical signs post-vaccination and could induce cross-protection against challenge with QX, a vaccination challenge experiment in SPF RIR chicks was conducted (Figure 1). Eight-day-old chicks were vaccinated with PBS, BeauR-4/91(S) or BeauR-M41(S), as per the grouping in Table 1. Clinical signs were not observed post-vaccination (Figure 2a) and the infectious vaccine virus was not recovered from the trachea at four days post-primary vaccination (dppv) (data not shown). Tracheal ciliary activity at four dppv in BeauR-4/91(S) and BeauR-M41(S) infected birds was comparable with the mock-vaccinated birds; the group means were 87%, 95%, and 87.5%, respectively (Figure 2b). These observations support previous reports that the S gene from a pathogenic isolate does not confer virulence to the apathogenic rIBV Beau-R [12,15,16]. 

### 3.2. Chickens Vaccinated with BeauR-M41(S) or BeauR-4/91(S) Exhibited Reduced Clinical Signs in Response to QX Challenge 

Fourteen days following primary vaccination, each group received a second vaccination of either BeauR-M41(S), BeauR-4/91(S), or PBS (as described in Table 1). The vaccination scheme included both homologous and heterologous boosts and, as previous research has indicated, vaccination order could influence the level of protection induced [25,49,50]. There were no clinical signs observed post-secondary vaccination (Figure 2c), further demonstrating the S gene from a pathogenic isolate cannot confer pathogenicity to a non-pathogenic strain.

**Figure 3:**
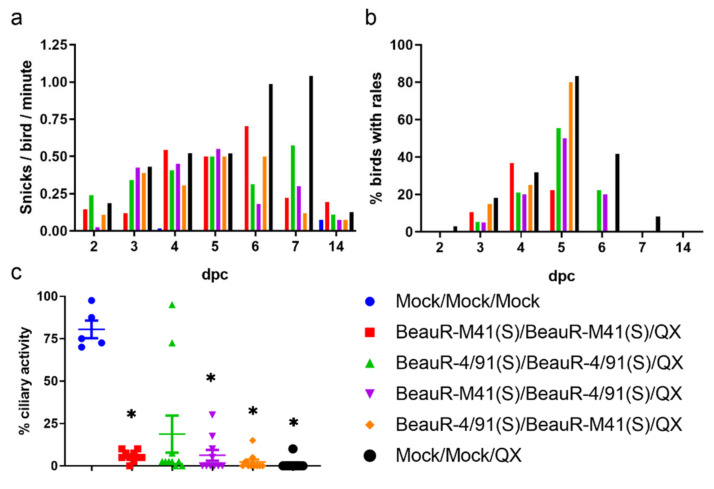
Vaccination with BeauR-M41(S) or BeauR-4/91(S) reduced the clinical signs observed after challenge with QX but was not able to protect against tracheal ciliostasis. Nine days post-secondary vaccination, birds were challenged with QX or mock challenged with PBS. (**a**) The numbers of snicks in each group were assessed from 2 to 7 dpc. Snicks were independently counted by two or three persons over a two-minute period with the average of these scores presented; (**b**) Birds were individually assessed for tracheal rales from 2 to 7 dpc. The percentage of birds per group positive for rales was calculated; (**c**) Trachea was harvested from 5 or 10 randomly sampled birds at 4 dpc. Each trachea was sectioned in 10 × 1 mm rings and the ciliary activity of each ring was assessed by light microscopy and the percentage activity calculated. Plotted points represent individual birds and the mean activity of the 10 rings assessed. Error bars represent SEM. Statistical differences were evaluated using a Kruskal–Wallis test followed by a post Hoc Mann–Whitney test corrected for multiple comparisons and are highlighted by * (*p* < 0.0001). Ciliary activity in all groups except BeauR-M41(S)/BeauR-4/91(S) was significantly reduced as compared with the mock vaccinated/mock challenged (Mock/Mock/Mock) group; ciliary activity in all vaccinated groups was comparable to the mock vaccinated/challenged (Mock/Mock/QX) control group.

Nine dpsv, birds were challenged with QX or mock challenged with PBS. From two to seven dpc, all groups, except those in the mock vaccinated/mock challenged control group (Mock/Mock/Mock), exhibited snicking and rales (Figure 3a,b). Until 5 dpc, there were no differences in both snicking rates and rales between all vaccinated/challenged groups and the mock vaccinated/challenged group (Mock/Mock/QX) (Figure 3a,b). At 6 dpc, the rate of snicking in the group vaccinated with BeauR-M41(S)/BeauR-M41(S) and the mock vaccinated/challenged control group (Mock/Mock/QX) increased, whereas in all other vaccinated groups it began to decrease. With the peak snicking rate in the mock vaccinated/challenged control birds (Mock/Mock/QX), one snick per bird per min, occurring at seven dpc as compared with the vaccinated/challenged groups (except for BeauR-4/91(S)/BeauR-4/91(S)) which exhibited less than 0.25 snicks per bird per min at the same time-point. At 14 dpc, snicking rates were comparable between all groups, indicating that, at this point, those birds challenged with QX had clinically recovered from the infection. The area under the curve (AUC) analysis using the trapezium rule indicates that the rates of snicking in the vaccinated groups were similar with mean values ranging from 2.48 for birds vaccinated with BeauR-4/91(S)/BeauR-M41(S) to 4.37 for birds vaccinated with BeauR-4/91(S)/BeauR-4/91(S). The mean AUC values of the snicking observed in the BeauR-M41(S)/BeauR-M41(S) and BeauR-M41(S)/BeauR-4/91(S) vaccinated groups were 3.51 and 3.08, respectively. In contrast, the mean AUC value for the snicking of the mock vaccinated/challenged control (Mock/Mock/QX) group was 7.16, and it was 0.28 for the mock vaccinated/mock challenged control group (Mock/Mock/Mock). The pattern of rales followed a similar trend, as vaccinated groups displayed lower percentages at six and seven dpc as compared with the mock vaccinated/challenged control (Mock/Mock/QX). Notably at seven dpc, rales were only observed in the mock vaccinated/challenged control group (Mock/Mock/QX) and, at six dpc, no rales were observed in birds vaccinated with BeauR-M41(S)/BeauR-M41(S) or BeauR-4/91(S)/BeauR-M41(S). The AUC analysis of the rales observed in the vaccinated groups ranged from mean values of 69.6 in the BeauR-M41(S)/BeauR-M41(S) vaccinated group to 120.0 in the BeauR-4/91(S)/BeauR-M41(S) vaccinated group. The rales observed in the BeauR-M41(S)/BeauR-4/91(S) and BeauR-4/91(S)/BeauR-4/91(S) groups had mean AUC values of 95.0 and 104.1, respectively, whilst the mock vaccinated/challenged control (Mock/Mock/QX) mean AUC value was considerably higher, at 209.9. There were no rales observed in the mock vaccinated/mock challenged control group (Mock/Mock/Mock). It was not possible to calculate whether these differences in mean AUC values were statistically significant due to the method used for the data collection (with a single value per group for each day), however, it appeared that with respect to clinical signs of snicking and rales, all vaccination regimes allowed the birds to recover quicker from the QX challenge infection.

### 3.3. Vaccination with BeauR-M41(S) or BeauR-4/91(S) Failed to Protect against Tracheal Ciliostasis Induced by a QX Heterologous Challenge

Tracheal ciliary activity was assessed in tracheas harvested at four dpc from ten randomly selected chickens per group except for the mock vaccinated/mock challenged group (Mock/Mock/Mock) in which five birds were assessed (Figure 3c). The industry standard for the assessment of IBV vaccines, set by the European Pharmacopeia, states that for a chicken to be classified as protected, at least 50% ciliary activity must be retained post-challenge in nine out of 10 tracheal rings [46]. The mean ciliary activity for the vaccinated groups ranged from 2.25%–18.75% (Table 2). Mock vaccinated/challenged control (Mock/Mock/QX) and the mock vaccinated/mock challenged control groups (Mock/Mock/Mock) exhibited 1% and 80.5% ciliary activity, respectively. Although there were some variations in the ciliary activity of the mock vaccinated/mock challenged control group such that only one bird had >90% ciliary activity, this did not alter the interpretation of vaccine effectiveness. Ciliary activity in the mock vaccinated/challenged group (Mock/Mock/QX) and the vaccinated groups, except BeauR-4/91(S)/BeauR-4/91(S), were significantly lower as compared with the mock vaccinated/mock challenged control (Mock/Mock/Mock) (*p* < 0.05). Interestingly, two birds within the BeauR-4/91/BeauR-4/91 vaccinated group retained ciliary activity comparable to mock vaccinated/mock challenged birds (Mock/Mock/Mock). One of these birds was classified as fully protected with 10 of 10 rings assessed retaining 90% or more ciliary activity (Table 2). The second bird retained this level of ciliary activity in seven of 10 rings assessed. 

### 3.4. Vaccination with a Heterologous Spike Protein Resulted in Reduced Viral Load in Homologous Boosted Vaccinated Groups after QX Challenge

**Figure 4:**
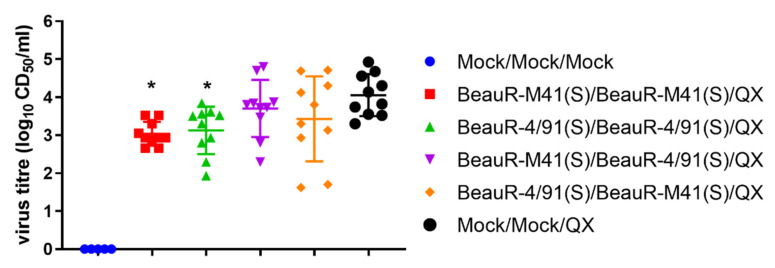
Vaccination with BeauR-M41(S)/BeauR-M41(S) and BeauR-4/91(S)/BeauR-4/91(S) resulted in a reduction in infectious viral load in response to the QX challenge within the trachea 4 dpc. Tissue-derived supernatant prepared from tracheas harvested at 4 dpc were titrated in ex vivo tracheal organ cultures (TOCs). Data points represent individual birds, with lines representing the mean and standard deviation (SD). Statistical differences between vaccinated groups and mock vaccinated/challenged control group (Mock/Mock/QX) highlighted by * (*p* < 0.05) were evaluated using a one–way ANOVA with a Tukey test for multiple comparisons.

To assess the implications of the vaccine combinations on infectious viral load, tissue-derived supernatant, prepared from tracheal rings harvested at four dpc, were titrated in ex vivo TOCs (Figure 4). There were no infectious virus isolated from tracheas harvested from the mock vaccinated/mock challenged birds (Mock/Mock/Mock). The tracheal viral loads were significantly lower (*p* < 0.05) in the two groups that received homologous primary and secondary vaccinations, i.e., BeauR-M41(S)/BeauR-M41(S) and BeauR-4/91(S)/BeauR-4/91(S) as compared with the mock vaccinated/challenged control group (Mock/Mock/QX). The tracheal viral loads of birds inoculated with heterologous primary and secondary vaccinations, BeauR-M41(S)/BeauR-4/91(S) and BeauR-4/91(S)/BeauR-M41(S), were comparable to the mock-vaccinated/challenged control group (Mock/Mock/QX). Interestingly, two birds from the BeauR-4/91(S)/BeauR-4/91(S) vaccinated group with post-challenge ciliary activity that was comparable with the mock controls, also had a comparable tracheal viral load with the group average (mean) (3.5, 3.8, and 3.13 log_10_ CD_50_/ml, respectively), and with the mock vaccinated/challenged control group (Mock/Mock/QX), at 4.06 log_10_ CD_50_/ml. Therefore, this observation could indicate that infectious viral load and ciliary activity, therefore, do not always necessarily correlate. 

**Figure 5:**
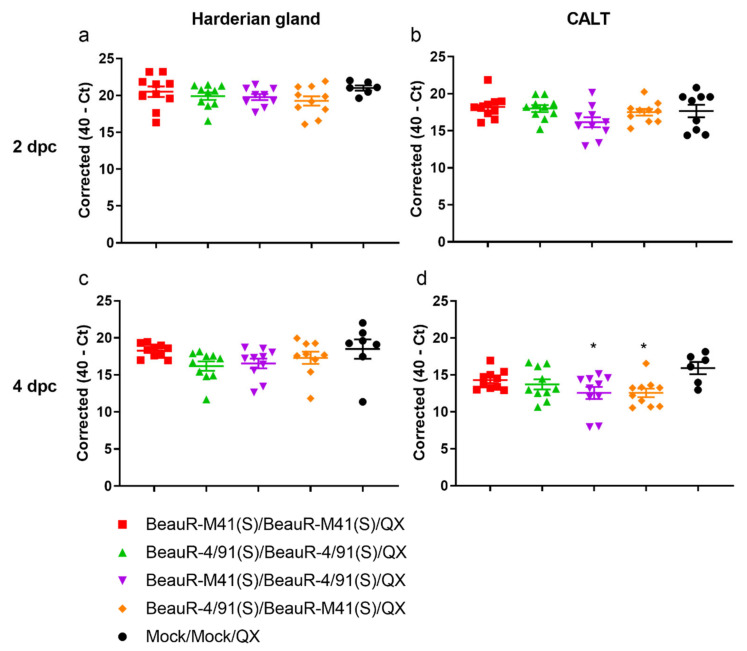
Vaccination with BeauR/M41(S)/BeauR-4/91(S) and BeauR-4/91(S)/BeauR-M41(S) resulted in a reduction in viral load in response to a QX challenge in the conjunctiva-associated lymphoid tissue (CALT) at 4 dpc. Relative viral RNA loads (expressed as corrected 40 cycle threshold) were assessed at specific time-points post-challenge. (**a**) Harderian gland at 2 dpc; (**b**) CALT at 2 dpc; (**c**) Harderian gland at 4 dpc; (**d**) CALT at 4 dpc. Data points are shown as the mean of three technical replicates per individual bird. Lines represent group mean and SEM. Statistical differences between the groups were evaluated using a one-way ANOVA with a TUKEY test for multiple comparisons. Significant differences between vaccinated groups and the mock vaccinated/challenged control group (Mock/Mock/QX) are highlighted by * (*p* < 0.05).

Viral RNA loads in the head-associated lymphoid tissues at two and four dpc were determined by RT-qPCR (Figure 5). At two dpc (Figure 5a,b), there were no significant differences identified between the vaccinated/challenged groups and mock vaccinated/challenged control group (Mock/Mock/QX). At four dpc in the CALT, but not in the Harderian gland (Figure 5c,d), a significant reduction in viral RNA load was detected in the groups which received different primary and secondary vaccinations, i.e., BeauR-M41(S)/BeauR-4/91(S) and BeauR-4/91(S)/BeauR-M41(S) (*p* < 0.05). However, there was no significant reduction in viral load in groups which received the same primary and secondary vaccination, i.e., BeauR-M41(S)/BeauR-M41(S) and BeauR-4/91(S)/BeauR-4/91(S), both of which had lower infectious viral load in the trachea (Figure 4). 

### 3.5. Vaccination with rIBV Induced the Production of IBV-Specific Antibodies and Primed Chickens for a Boosted Humoral Response to Challenge

**Figure 6:**
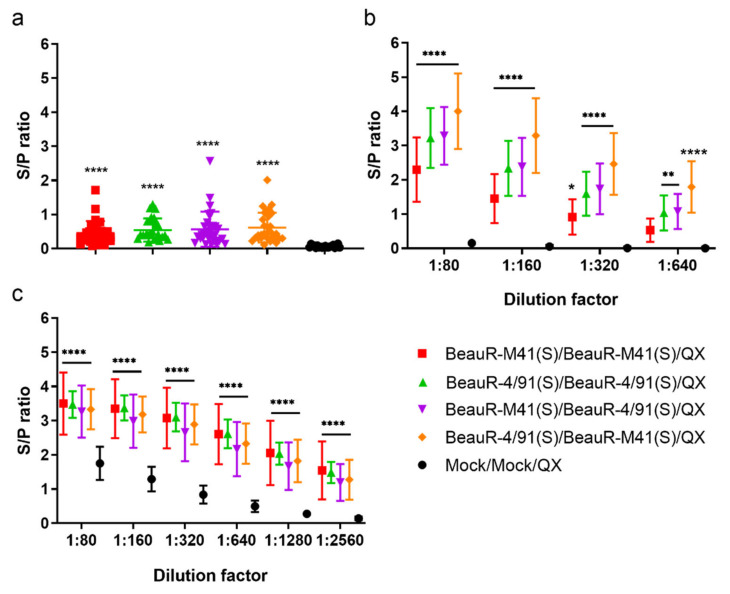
Vaccination with recombinant infectious bronchitis virus (rIBV) BeauR-M41(S) and BeauR-4/91(S) induced the production of IBV-specific antibodies and primed chickens for a boosted humoral response to challenge. Serum anti-IBV antibody titres were assessed by commercial ELISA (BioCheck, Reeuwijk, The Netherlands). (**a**) Pre-challenge serum samples were diluted 1:80 and dilutions 1:80 through to 1:2560 were investigated for (**b**) 4 dpc serum samples and (**c**) 14 dpc serum samples. The mean S/P of four technical replicates of each bird from each group is presented. The cut-off threshold for positive samples is S/P ratio = 0.2. The error bars represent SD. Statistical differences between the group antibody titre means at (a) pre-challenge were assessed using Kruskal–Wallis and Dunn’s multiple comparison test. Post-challenge (b) and (c), antibody titres were assessed using a one-way ANOVA with a Friedman test and Dunn’s multiple comparison test. Significant differences between vaccinated groups and the mock vaccinated/challenged (Mock/Mock/QX) control group are highlighted by * (*p* < 0.05), ** (*p* < 0.01), and **** (*p* < 0.0001).

Serum IBV-specific antibodies were assessed post-vaccination, at seven dpsv, and post-challenge, at four and 14 dpc, and compared with the mock vaccinated/challenged (Mock/Mock/QX) control (Figure 6). At seven dpsv (pre-challenge), mean anti-IBV titres were significantly higher in all the vaccinated groups as compared with the mock vaccinated/mock challenged (Mock/Mock/Mock) control (*p* < 0.0001, Figure 6a), demonstrating the vaccination regimes employed had induced IBV-specific serum antibodies. However, there were no significant differences across the vaccinated groups pre-challenge. At four dpc, serum titres were significantly higher in the vaccinated groups as compared with the mock vaccinated/challenged control group (Mock/Mock/QX) (Figure 6b, *p* < 0.05), indicating that the rIBV vaccination primed the birds for challenge and induced strong humoral response, as shown previously [12]. Interestingly, there were significant differences in the capability of the rIBV vaccines expressing different S proteins to induce IBV specific responses. The highest titres were induced by a heterologous vaccination with BeauR-4/91(S) followed by BeauR-M41(S). When the birds were vaccinated in the reverse order, i.e., BeauR-M41(S)/BeauR-4/91(S), the titres were significantly lower (*p* < 0.0001). Vaccination with BeauR-M41(S)/BeauR-4/91(S) or with BeauR-4/91(S)/BeauR-4/91(S) induced similar titres, whereas vaccination with BeauR-M41(S)/BeauR-M41(S) induced the lowest titres (Figure 6b). Collectively, this indicates that the order of vaccination can impact the magnitude of humoral responses induced to rIBV. Serum titres in the mock vaccinated/challenged (Mock/Mock/QX) control group remained below detection level. At 14 dpc, serum antibody titres were still significantly higher in all vaccinated groups as compared with the mock vaccinated/challenged (Mock/Mock/QX) control group (Figure 6c, *p* < 0.0001), providing evidence of a boosted antibody response in vaccinated groups in response to the challenge with a heterologous strain, QX. At 14 dpc, there were no significant differences between the anti-IBV titres in the vaccinated groups (Figure 6c).

## 4. Discussion

In this study, vaccination with BeauR-M41(S) or with BeauR-4/91(S) in either homologous or heterologous booster regimes, did not induce a fully protective immune response against the heterologous QX challenge as defined by the European Pharmacopeia as protection against ciliostasis [46]. Ciliary activity in all vaccinated groups post-challenge was reduced to less than 25% (Figure 3). Interestingly, two birds that were vaccinated with BeauR-4/91(S)/BeauR-491(S) were observed to retain ciliary activity post-challenge, however, only one bird could be deemed to be protected in accordance with European Pharmacopeia [46] standards (Table 2). However, despite the overall reductions in ciliary activities observed in all the vaccinated groups, there were indications that each of the vaccination strategies induced an immune response that resulted in positive differences post-challenge. All vaccinated groups appeared to recover more rapidly from clinical signs than the mock vaccinated/challenged (Mock/Mock/QX) control group (Figure 3). The homologous vaccinated groups, BeauR-M41(S)/BeauR-M41(S) and BeauR-4/91(S)/BeauR-4/91(S), were the only groups that had reduced infectious tracheal viral load, at four dpc as compared with the mock vaccinated/challenged (Mock/Mock/QX) group (Figure 4). The heterologous primary and secondary vaccination, BeauR-M41(S)/BeauR-4/91(S) or BeauR-4/91(S)/BeauR-M41(S), was associated with lower viral RNA loads in the CALT, at four dpc as compared with the mock vaccinated/challenged (Mock/Mock/QX) control group. In addition, all vaccinated groups at seven dpsv and four dpc had higher titres of serum anti-IBV antibody than the mock vaccinated/challenged (Mock/Mock/QX) control group, demonstrating that the vaccination had induced a humoral response (Figure 6). Unfortunately, whether these humoral responses included neutralising antibody toward QX remains an unanswered question due to the inability to propagate the QX strain used in this study in vitro [14]. 

Numerous studies have addressed the question of cross-protective immunity against IBV and have reported varying degrees of success [21,22,24,25,49,51]. The S glycoprotein is the main inducer of protective immunity [8,9,52]. It is thought that the chances of a successful vaccination decrease as the amino acid homology between the S glycoprotein of the vaccine and challenge strain also decreases [22,49]. As homology decreases, the likelihood of differences arising in important neutralising epitopes increases. It is possible, however, for vaccines to offer successful protection against heterologous challenge when sequence homology between the vaccine and challenge strain is deemed to be low (as reviewed in [26]), presumably due to conserved structural homology of epitopes involved in viral neutralisation. A small number of virus neutralising epitopes are likely to play a disproportionate role in cross-protection [22]. Although the locations of some neutralising monoclonal antibody epitopes have been identified [53,54,55], it remains unknown which epitopes are required for the successful induction of cross-protective immunity. Therefore, it continues to be important to investigate and assess the level of protection afforded by vaccine strategies against heterologous challenge.

Several previous studies have investigated cross-protective immunity utilising commercial vaccines produced via the traditional method of passaging a field isolate through embryonated hens’ eggs to produce an attenuated virus [21,25,49,50]. As such, these commercial vaccines closely resemble the challenge strains, and therefore it is difficult to assess the individual contribution of each viral protein towards both homologous and heterologous immunity. The recombinant viruses used in this study, also used by Hodgson et al. (2004), Armesto et al. (2011), and Ellis et al. (2018), express heterologous S glycoproteins from a rIBV vector [12,15,16]. As the other proteins in the vaccine viruses are Beaudette derived, the utilisation of BeauR-M41(S) and BeauR-4/91(S) vaccines allowed for characterisation of the specific role of the 4/91 and M41-CK S glycoproteins in induction of a protective immune response against a heterologous QX challenge. Amino acid sequence identity among E, M, and N of QX and Beau-R is relatively low at 88%, 92%, and 90%, respectively, and additionally, the Beaudette strain is considered to be a weak stimulator of humoral immune responses in vivo [15,56]. In previous studies that have investigated vaccination with both Massachusetts and 4/91 serotypes against a QX challenge, it has been difficult to ascertain whether the protective response induced is solely the result of the presence of the S glycoprotein of either the Massachusetts strain or 4/91 strain [21,26,50]. In this study, we used rIBVs to address the role of the spike glycoprotein in cross-protection, along with an analysis of whether the order of heterologous vaccination impacted cross-protection.

The order in which live attenuated IBV vaccines are administered has been shown to affect the outcome of heterologous challenge. Cook et al. (1999) concluded that better cross-protection was seen when a Massachusetts serotype vaccine, Ma5, was administered before a 4/91 vaccine [49]. Additionally, Awad et al. (2016) and Bru et al. (2017) demonstrated a degree of cross-protection using a Massachusetts vaccine followed by a secondary vaccine of a different serotype, however, the reverse order was not directly compared [25,50]. Whilst in terms of ciliary activity, there was no difference between those birds vaccinated with BeauR-M41(S)/BeauR-4/91(S) and BeauR-4/91(S)/BeauR-M41(S) in this study, the order in which the BeauR-M41(S) (Massachusetts serotype) and BeauR-4/91(S) (4/91 serotype) were administered did appear to have affected serum antibody levels. Serum antibody titres assessed at four dpc were significantly higher in the group vaccinated with BeauR-4/91(S)/BeauR-M41(S) as compared with the other vaccination regimes, suggesting, in this study, that a 4/91 vaccine followed by a Massachusetts vaccine could have enhanced the level of seropositivity. 

Despite the indication that the vaccination strategies employed in this study induced an immune response, ultimately the vaccines did not offer protection against ciliostasis following a QX challenge, which is the gold standard method of assessing protection. This was unexpected as it contradicts previous work that has demonstrated that commercially available Massachusetts and 4/91 vaccines confer protection against a heterologous QX challenge [21,50,57]. Awad et al. (2016) investigated combinations of Ma5 or H120, both of the Massachusetts serotype, alongside 793B vaccines (4/91 serotype) against heterologous QX challenge; ciliary activity was reported at 68% and 92%, respectively [50]. Similarly, de Wit et al. (2011) used a combination of Massachusetts vaccines or a mixture of Massachusetts combined with a D274 serotype, followed by a 793B vaccine and reported protection, as defined by ciliary activities of 51% and 89%, respectively [26]. Terregino et al. (2008) also evaluated a vaccination with Ma5 and 4/91, against a QX challenge [21]. Unlike this study in which challenge virus was detected in tracheas from all vaccinated/challenged chickens, Terregino et al. (2008) isolated no virus post-challenge in vaccinated SPF chickens. However, it is difficult to make direct comparisons between vaccine-challenge experiments due to differences in the range of parameters which include age, breed, SPF status, and the basis for defining protection (ciliary activity, viral load or seropositivity) (reviewed by [58,59]). Terregino et al. (2008) did not assess ciliary activity and it has been well documented that viral load and ciliary activity do not always correlate [12,15,16,58]. Similarly, seropositivity and protection do not always correlate (reviewed by [58]). A recent study into correlates of IBV-vaccine-induced protection revealed that birds with the highest monocyte major histocompatibility complex (MHC) II expression had the weakest vaccine-induced protection [60]. Whilst de Wit et al. (2011) assessed ciliary activity at 5, 8, and 11 dpc, these sampling time-points were not directly comparable to the time-point, four dpc, used here [26]. In addition, there were no clinical signs data reported, and the age of the chickens, at both vaccination and challenge, were also not comparable. It is also worth noting that the vaccinated birds in both studies by both Terregino et al. (2008) and Awad et al. (2016), were vaccinated in the presence of maternally-derived antibody, although this is thought to have a negative impact on vaccination [61]. The inability of BeauR-M41(S) and BeauR-4/91(S) to induce a protective immune response against heterologous virus challenge, in terms of both ciliary activity (Figure 2c) and challenge virus replication (Figure 2) is a notably different result to that published by [21,50,57]. This raises some interesting questions and could indicate that the S glycoprotein from either M41-CK and/or 4/91 alone expressed by a Beaudette backbone may not be sufficient to elicit an adequately protective immune response against a heterologous QX challenge.

The other possibility is the limited replication of the vaccine viruses in vivo, since as observed in previous studies [12,15,16], neither rIBV BeauR-M41(S) nor BeauR-4/91(S) could be detected in the trachea post-vaccination (data not shown). The rIBV Beau-R, the genetic backbone of the vaccine viruses used here, is a molecular clone of the apathogenic Beaudette-CK strain [38]. Ellis et al. (2018) investigated the ability of Beau-R expressing heterologous S1 or S2 subunits to induce protective immune responses against homologous challenge [12]. Whilst rIBV BeauR-M41(S) induced partial protective immunity, as also reported by Hodgson et al. (2004) [15], the response was not robust. In addition, ciliary activity, post-challenge (65%), fell short of the European Pharmacopeia standards for full protection, a finding comparable with both Hodgson et al. (2004) [15], and Armesto et al. (2011) who investigated homologous and heterologous challenge using BeauR-4/91(S) [16]. It has long been established that the Beaudette strain cannot establish a long-lasting productive infection in vivo, with the molecular clone Beau-R behaving similarly [15,56]. Interestingly, chickens inoculated with Beaudette do produce virus neutralising antibodies, however, these rapidly decrease over time [56], presumably due to the lack of viral replication.

## 5. Conclusions

In summary, this study demonstrates that vaccination with rIBV BeauR-M41(S) and BeauR-4/91(S) can induce anti-IBV humoral responses, however, these responses have not translated into protection against ciliostasis following a heterologous challenge. The inability of the rIBV vaccines to elicit a stronger protective immune response has raised several questions that are critical to answer to enable the design of the next generation of rationally attenuated vaccines. It is important to establish whether a less attenuated IBV vaccine backbone, with improved replication in vivo, would induce a protective immune response to both homologous and heterologous challenges, meeting the criteria defined by the European Pharmacopeia [46]. Additionally, it is relevant to explore whether replacement of other structural proteins (E, M, or N) in the rIBV Beau-R backbone, with those derived from pathogenic strains, improves the induction of an effective cross-protective immune response. Further work is required to identify the immunogenic epitopes presented on the S glycoproteins from different IBV serotypes to elucidate whether rationally designed vaccines expressing both cross-reactive epitopes and serotype-specific epitopes are capable of inducing cross-neutralising antibodies, resulting in the generation of novel cross-protective IBV vaccines. 

## Figures and Tables

**Table 1:** Details of groups, vaccination schedule, sampling points, and bird numbers.

Primary Vaccination	No. of Birds Sampled	Secondary Vaccination	Challenge	No. of Birds Sampled	Total no. of Birds Per Group
Mock	5 birds on 4 dppv	Mock	Mock	5 birds on 2, 4 and 14 dpc	20
BeauR-M41(S)	5 birds on 4 dppv	BeauR-M41(S)	QX	10 birds on 2 and 4 dpc; 9 * birds on 14 dpc	35
BeauR-4/91(S)	5 birds on 4 dppv	BeauR-4/91(S)	QX	10 birds on 2 and 4 dpc; 9 birds on 14 dpc	34 ^#^
BeauR-M41(S)	0	BeauR-4/91(S)	QX	10 birds on 2, 4 and 14 dpc	30
BeauR-4/91(S)	0	BeauR-M41(S)	QX	10 birds on 2, 4 and 14 dpc	30
Mock	0	Mock	QX	10 birds on 2 and 4 dpc; 12 birds on 14 dpc	32

Abbreviations: dppv, days post-primary vaccination; dpc, days post-challenge. *, one bird died unexpectedly post-vaccination and the cause of death was not related to infectious bronchitis virus (IBV); ^#^, one bird died before the study started and the cause of death was not related to IBV.

**Table 2:** Assessment of protection against ciliostasis associated with BeauR-M41(S) and BeauR-4/91(S) vaccination following challenge with QX.

Primary Vaccination/ Secondary Vaccination/ Challenge	% Ciliary Activity (Mean ± SD)	No. of Birds with 90% Ciliary Activity/Total No. of Birds Examined	% of Group Protected
Mock/Mock/Mock	80.50 ± 11.65	1/5	N/A
BeauR/M41(S)/BeauR-M41(S)/QX	5.75 ± 3.13	0/10	0
BeauR-4/91(S)/BeauR-4/91(S)/QX	18.75 ± 34.73	1/10	10
BeauR-M41(S)/BeauR-4/91(S)/QX	6.25 ± 10.22	0/10	0
BeauR-4/91(S)/BeauR-M41(S)/QX	2.25 ± 4.78	0/10	0
Mock/Mock/QX	1.00 ± 3.16	0/10	0
